# Optima Nutrition: an allocative efficiency tool to reduce childhood stunting by better targeting of nutrition-related interventions

**DOI:** 10.1186/s12889-018-5294-z

**Published:** 2018-03-20

**Authors:** Ruth Pearson, Madhura Killedar, Janka Petravic, Jakub J. Kakietek, Nick Scott, Kelsey L. Grantham, Robyn M. Stuart, David J. Kedziora, Cliff C. Kerr, Jolene Skordis-Worrall, Meera Shekhar, David P. Wilson

**Affiliations:** 1 Burnet Institute, Melbourne, Australia; 2 Department of Epidemiology and Preventive Medicine, Monash University, Melbourne, Australia; 3 Optima Consortium for Decision Science, Melbourne, Australia; 4 The World Bank, ICF International, Washington D.C., USA; 5 Department of Mathematical Sciences, University of Copenhagen, Copenhagen, Denmark; 6 Complex Systems Group, School of Physics, University of Sydney, Sydney, Australia; 7 Institute for Global Health, University College London, London, UK

## Abstract

**Background:**

Child stunting due to chronic malnutrition is a major problem in low- and middle-income countries due, in part, to inadequate nutrition-related practices and insufficient access to services. Limited budgets for nutritional interventions mean that available resources must be targeted in the most cost-effective manner to have the greatest impact. Quantitative tools can help guide budget allocation decisions.

**Methods:**

The Optima approach is an established framework to conduct resource allocation optimization analyses. We applied this approach to develop a new tool, ‘*Optima Nutrition’,* for conducting allocative efficiency analyses that address childhood stunting. At the core of the Optima approach is an epidemiological model for assessing the burden of disease; we use an adapted version of the *Lives Saved Tool* (LiST). Six nutritional interventions have been included in the first release of the tool: antenatal micronutrient supplementation, balanced energy-protein supplementation, exclusive breastfeeding promotion, promotion of improved infant and young child feeding (IYCF) practices, public provision of complementary foods, and vitamin A supplementation. To demonstrate the use of this tool, we applied it to evaluate the optimal allocation of resources in 7 districts in Bangladesh, using both publicly available data (such as through DHS) and data from a complementary costing study.

**Results:**

Optima Nutrition can be used to estimate how to target resources to improve nutrition outcomes. Specifically, for the Bangladesh example, despite only limited nutrition-related funding available (an estimated $0.75 per person in need per year), even without any extra resources, better targeting of investments in nutrition programming could increase the cumulative number of children living without stunting by 1.3 million (an extra 5%) by 2030 compared to the current resource allocation. To minimize stunting, priority interventions should include promotion of improved IYCF practices as well as vitamin A supplementation. Once these programs are adequately funded, the public provision of complementary foods should be funded as the next priority. Programmatic efforts should give greatest emphasis to the regions of Dhaka and Chittagong, which have the greatest number of stunted children.

**Conclusions:**

A resource optimization tool can provide important guidance for targeting nutrition investments to achieve greater impact.

## Background

Stunting, or reduced linear growth, affects around 200 million children younger than 5 years of age – mostly in Asia and Africa [1, 2]. Stunting is a predictor of poorer cognitive and physical development, which has been shown to reduce productive capacity and economic output [3–8]. Sustainable Development Goal 2 calls for an end to malnutrition by 2030 and significant reductions in stunting by 2025. Research suggests that this goal can be partly met through a set of evidence-based interventions [9] in the period from conception to 5 years of age [10].

There are numerous proven ways in which undernutrition and stunting can be reduced. Correct feeding practices, which include exclusive breastfeeding for the first 6 months and continued breastfeeding, combined with appropriate complementary foods until the age of 24 months, not only provide a key source of nutrients but also protection against gastrointestinal and other infections [11]. Sub-optimal breastfeeding is associated with poorer child growth outcomes because of replacement with less nutritious foods and greater risk of infection [12, 13]. For children aged 6 months to 5 years, providing high quality and nutritionally diverse complementary foods, and supplementing diets with micro-nutrients including vitamin A and zinc, if needed, can also reduce the risk of stunting [14–16]. In addition to nutritional interventions, the risk of stunting can be reduced by addressing household, environmental, socioeconomic and cultural factors [17, 18]. The quality and use of health, social protection, and sanitation service delivery systems are further elements affecting nutrition outcomes.

Since there are only scarce resources from domestic sources or from international donors for nutrition-related interventions [19], it is imperative that available budgets are used in a manner which can achieve the greatest possible outcome. There is evidence from diverse settings that better health resource allocation decisions result in substantial improvements in population health [20–22]. Quantitative tools can be useful to integrate available knowledge and data into a logical framework and provide analytical evidence for improving resource allocation decisions. This study introduces a new tool, Optima Nutrition, specially designed and developed to inform allocative efficiency decisions related to stunting.

Existing modeling tools provide an effective base for enabling policy makers to estimate the epidemiological impact of nutritional interventions on population outcomes. These include the *Lives Saved Tool* (LiST) [23, 24], PROFILES [25], World Breastfeeding Costing Initiative (WBCi) [26], CMAM Costing Tool [27] and MINIMOD [28]. LiST calculates the expected impact and cost of scaling up numerous maternal and child health interventions including nutritional interventions; PROFILES demonstrates the effect of nutritional interventions on health and economic development; and WBCi estimates the cost and impact of scaling up promotion and support services for breastfeeding and correct infant and young child feeding (IYCF) practices. The CMAM Costing Tool estimates the cost of establishing, maintaining or expanding services for community management of acute malnutrition (CMAM), and is offered as a part of FANTA (Food and Nutrition Technical Assistance) Project [29] – a USAID-funded project for strengthening food security and nutrition policies. MINIMOD finds the mix of delivery systems that maximizes effective coverage of a micronutrient supplementation. Some of these tools consider the benefits of single interventions, whereas others incorporate the effects of multiple interventions simultaneously. Currently, no tool is capable of estimating the allocation of a given budget across a range of nutritional interventions that gets as close as possible to attaining certain objectives. The Optima Nutrition tool aims to fill this gap by building on the strengths of the existing nutrition modeling field and expertise in allocative efficiency tools from other fields.

A consortium of academic institutions and development partners has produced a suite of modeling tools with central focus on improving allocative efficiency. Optima tools have been applied in partnership with the governments of over 40 low- and middle-income countries to assess how more targeted resource allocation decisions can lead to improved outcomes for major infectious diseases including HIV, TB and malaria (e.g. [30–35])). These studies have influenced health resource allocation, shifting funding to the most cost-effective mix of programs and assisting with national strategic plan development and operational planning in many countries.

## Methods

The Optima approach combines: (i) a core epidemiological model that relates intervention outcomes to epidemiological outcomes; (ii) cost functions that relate coverage and expenditure on interventions to intervention outcomes; (iii) an objective function defined by national strategic targets and constraints defined by logistic, ethical, political and financial considerations; (iv) a formal mathematical optimization algorithm around other components to estimate the most allocatively efficient use of resources. To develop the Optima Nutrition tool, we followed this approach, using a replicated version of the LiST model to serve as the core epidemiological model. Both LiST and the Optima approach have been described in detail elsewhere [24, 30], but in this section we provide a short summary of them both, and describe how they were integrated to develop Optima Nutrition. In addition, we provide details on the data that were collated and used in the demonstration example of Optima Nutrition’s application to Bangladesh.

### Epidemiological model structure

**Fig. 1:**
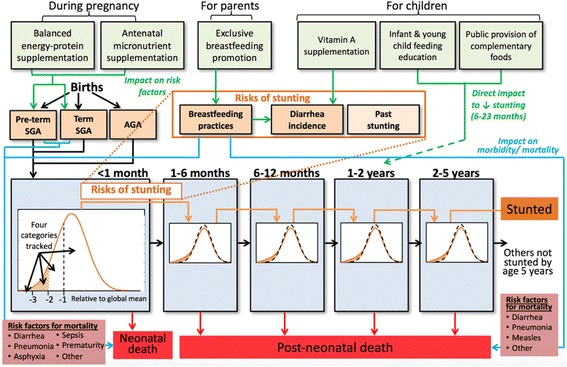
Diagram of the Optima Nutrition model. The number of children under 5 years is tracked across five age bands. Children enter the model by birth: term or pre-term, small for gestational age (SGA) or average for gestational age (AGA). Children leave the model either by reaching 5 years of age, or by death. Risk factors which affect stunting and mortality, and interventions included in the model to reduce mortality and stunting, are shown

The core epidemiological model used within Optima Nutrition is a dynamic, deterministic, compartmental model which tracks the number of children in a population from birth until 5 years of age across five age bands: < 1 month, 1–5 months, 6–11 months, 12–23 months and 24–59 months (see Fig. 1). Children enter the model cohort via the < 1 month age-band at birth and exit the cohort either when they reach the age of 60 months or by death, which can happen at any age. Children in each age-band are categorized by height-for-age and breastfeeding practice. The number of children in each category is simulated as they age through, and out of, the modeled population, estimating overall stunting and mortality. Risk factors for stunting and death are: birth outcomes including pre-term birth or a child being born small for gestational age, stunting in the previous age-band, suboptimal breastfeeding practices, and incidence of diarrhea (Fig. 1). In the model, children in the < 1 month age-band can die due to diarrhea, pneumonia, meningitis, asphyxia, sepsis, prematurity and “other” causes, while children in all other age bands can die of diarrhea, pneumonia, measles and “other” causes. The relative risks of dying from each cause are related to the child’s breastfeeding and height-for-age status. Risks of stunting and mortality associated with these risk factors are taken from established literature [36–38]; “other” causes are used to capture, and match to, population statistics of known overall mortality rates for the given application context. The model equations, along with mathematical expressions that link parameter values from the literature to these model rates, are the same as used in LiST and provided in [23].

The model divides children in each age-band into four height-for-age categories: more than 3 standard deviations below the global norm (‘severely stunted’), 2–3 standard deviations below the norm (‘moderately stunted’), 1–2 standard deviations below the norm (‘mildly stunted’, and less than one standard deviation below the norm (‘normal’). Children in all age bands who are more than two standard deviations below the global norm are considered stunted. Breastfeeding practice is categorized as none, partial (liquids and solids in addition to breastmilk), predominant (breastmilk supplemented by other liquids), and exclusive.

### Interventions included in optima nutrition

Six nutritional interventions that target child mortality and stunting are included in this application of the model: antenatal micronutrient supplementation (AMS), balanced energy-protein supplementation during pregnancy, promotion of exclusive breastfeeding, promotion of improved infant and young child feeding (IYCF) practices, public provision of complementary foods and vitamin A supplementation.

The six interventions included in this release of the Optima Nutrition tool either directly impact mortality and the risk of stunting in different age bands, or have influence by impacting associated risk factors including incidence of diarrhea, breastfeeding practice or birth outcomes. The impact of these interventions on different age bands in the modeled cohort is illustrated in Fig. 1:

- Antenatal micronutrient supplementation – given to pregnant women, improves birth outcomes [39]
- Balanced protein-energy supplementation – given to pregnant women below poverty line, improves birth outcomes [40].
- Breastfeeding promotion – behavioral intervention that targets pregnant women and (mothers of) children < 6 months, increases prevalence of exclusive breastfeeding of children under 6 months of age, which reduces incidence of diarrhea and all-cause mortality in this age group [37, 41].
- Infant and young child feeding program – behavioral intervention targeting (mothers of) children 6–23 months of age, which reduces stunting and all-cause mortality by promoting partial breastfeeding and correct complementary feeding practices. Complementary feeding education can only benefit the population with sufficient income to afford recommended complementary foods [42].
- Public provision of complementary foods – complementary food provided to children 6–23 months below poverty line in addition to complementary feeding education, in order to reduce their risk of stunting. We assume that education around complementary feeding does not impact the population below the poverty line unless supplemented with public provision of complementary foods [43].
- Vitamin A supplementation – given to children 6 months and older to reduce the incidence of and mortality from diarrhea [44, 45].

As a form of validation of the epidemiological component of our model, we compared the effects of scaling up the included interventions on stunting and mortality to what was estimated by LiST. For the number of child deaths per year, Optima and LiST are in good agreement, with the same percentage of averted deaths. There are small differences between the predictions of stunting averted in the two models (Optima has a slightly higher estimate), which can be explained by the differences in updating timesteps (yearly in LiST, monthly in Optima).

### Cost functions and resource optimization framework

**Fig. 2:**
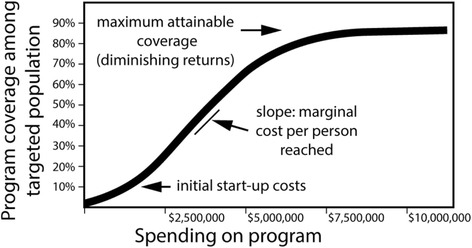
Generic form of a cost function associated with an intervention program used in an Optima model

The Optima approach links the core epidemiological model to a resource optimization algorithm through cost functions related to each intervention. Cost functions are a key driver of resource optimization and encapsulate the set of relationships between (a) the cost of service delivery, (b) the resulting coverage levels of these services among targeted populations, and (c) how these coverage levels of services influence behavioral, clinical and epidemiological outcomes. Such relationships dictate how incremental changes in spending directly or indirectly affect outcomes of interest or associated risk factors. The cost functions can take any functional form. Most typically, a 3-parameter logistic (sigmoidal) function is fitted to available historical expenditure data, or a 2-parameter logistic function is parameterized using unit costs and information about logistical or feasibility constraints for program coverage levels. A schematic illustration of a cost function is shown in Fig. 2. The cost functions combined with the epidemiological model can project the expected level and trend in number of stunted children and number of deaths in the context of different intervention programs operating together at various funding and coverage levels.

The next step in resource optimization is to clearly define the objective using an objective function. Different objective functions will lead to different resource allocations and consequently different outcomes. For example, the objective to solely minimize the number of stunted children at age 5 years and the objective to solely minimize the number of child deaths would lead to different funding allocations and have conflicting outcomes. Minimizing cases of stunting would require preferential funding of the nutrient-intake interventions that have little direct impact on general survival (such as complementary feeding), which may result in increased mortality, particularly in stunted children. On the other hand, minimizing child deaths would preferentially fund interventions with direct impact on mortality (such as breastfeeding promotion), and may increase stunting prevalence because of improved survival of stunted children. We explored numerous objective functions (any of which could easily be used in the model) but have established a default objective function for Optima Nutrition of maximizing the number of children who are alive and not stunted by age 5 years (the ‘alive and not stunted’ objective). Although any time horizon can be used in analyses, the default time horizon for Optima Nutrition is from the next year of programming (e.g. 2018) until 2030, the end of the sustainable development goals (SDGs).

Many optimization algorithms could be used around the calibrated epidemiological model informed by setting-specific data, and the cost functions, to calculate the resource allocation that will be the best solution against the objective function. Optima Nutrition uses an Adaptive Stochastic Descent (ASD) algorithm [46]. ASD uses simple principles to form probabilistic assumptions about (a) which parameters have the greatest effect on the objective function, and (b) optimal step sizes for each parameter.

### A demonstration of optima nutrition: Bangladesh as an illustrative case study

We demonstrate the use of Optima Nutrition through an example application. For illustrative purposes, the model is populated with available data from Bangladesh. Bangladesh has a population of about 160 million, increasing at 2 million (1.25%) annually. While the under-five mortality rate has dropped considerably over the last 25 years, it is currently estimated at 46 deaths per 1000 live births, of which half are neonatal. Of the approximately 15 million children aged less than 5 years in Bangladesh, over 5 million (36%) are stunted [47]. This high stunting prevalence is contributing to relatively very high child mortality as well as reduced cognitive development and related consequences [48].

The epidemiological model within Optima Nutrition was populated with available data from seven administrative divisions of Bangladesh (Barisal, Chittagong, Dhaka, Khulna, Rajshahi, Rangpur and Sylhet). Demographic data (population size in each of the five age-groups) was taken from the Bangladesh Bureau of Statistics [49], and the 2016 number and future projections of live births were taken from the UN Population Division database. Child mortality and prevalence of stunting, breastfeeding practices in each age group and poverty data were taken from the Bangladesh Demographic and Health Surveys [50, 51] Unit cost data to inform cost functions was taken from a report led by the World Bank and UNICEF for the Government of Bangladesh [52]. These unit costs were calculated using program experience approach, which captures all aspects of service delivery—including the costs of commodities, transportation and storage, personnel, training, supervision, monitoring and evaluation, relevant overhead, wastage, etc. for each intervention. We assume that the relationship between investment and the number of people reached by each intervention is approximately linear at low and moderate coverage levels, but that marginal costs steadily increase at higher coverage levels (see Fig. 2) as the remainder of the target population may be hard to reach [53]. We have not accounted for any start-up costs in this application because of insufficient data and because recurrent annual costs are most important in the optimization of the annual budget. For each of the interventions considered, the estimated national 2014 coverage levels and unit costs are provided in Table 1. Region-specific Unit costs were the same as national for all interventions except AMS, which was the most expensive in Rangpur ($1.82) and the least expensive in Dhaka ($1.78). The unit costs are assumed to be the marginal costs at low program coverage, which is applicable for current scale of programs in Bangladesh.

**Table 1:** Interventions to target stunting [9] and their baseline coverage in Bangladesh

Maternal interventions	Target population	Description & impact	Estimated program model inputs for the national Bangladesh illustration		
			Coverage	Unit cost (marginal cost at low coverage)	2014 spending on program
Balanced energy-protein supplementation	Pregnant women in the lowest two wealth quintiles	Improves birthweight, reduction in incidence of infants born small for gestational age	0%	$25.00	$0
Antenatal micronutrient supplementation	Pregnant women	Improves birthweight, reduction in incidence of infants born small for gestational age	0%	$1.80	$0
Child interventions					
Exclusive breastfeeding promotion	Pregnant women and mothers of children under 6 months	Breastfeeding decreases mortality due to other causes & decreases stunting by decreasing incidence of diarrhea	61% Indicator: *% children < 6 months who are exclusively breastfed*	$3.56	$14.3 m
Infant & young child feeding (IYCF) education	Children 6–23 months old	Direct impact on stunting, must be combined with CFS to benefit food insecure	24.7% Indicator: *% mothers of children > 5 months with 4 or 4+ visits*	$3.56	$4.1 m
Public provision of complementary foods (CFS)	Children 6–23 months old, food insecure	Direct impact on stunting, benefits only food insecure	0%	$48.00	$0
Vitamin A supplementation	Children 6–59 months old	Decreases mortality due to diarrhea & decreases stunting by decreasing diarrhea incidence	62.1%	$0.35	$3.8 m

For each of the seven administrative divisions, we estimated the optimal distribution of resources across intervention programs under a range of different total available budgets. This provided a relationship between total available budget and number of stunted children and/or child deaths for each geographical region, representing the best available outcome attainable for each budget. This ‘budget-outcome’ relationship was used with interpolations to generate a curve for a predicted projection of the best available outcome attainable for any budget. By comparing the budget-outcome curves between geographical regions, the optimization algorithm was extended to optimize national outcomes by shifting funding between divisions.

## Results

**Fig. 3:**
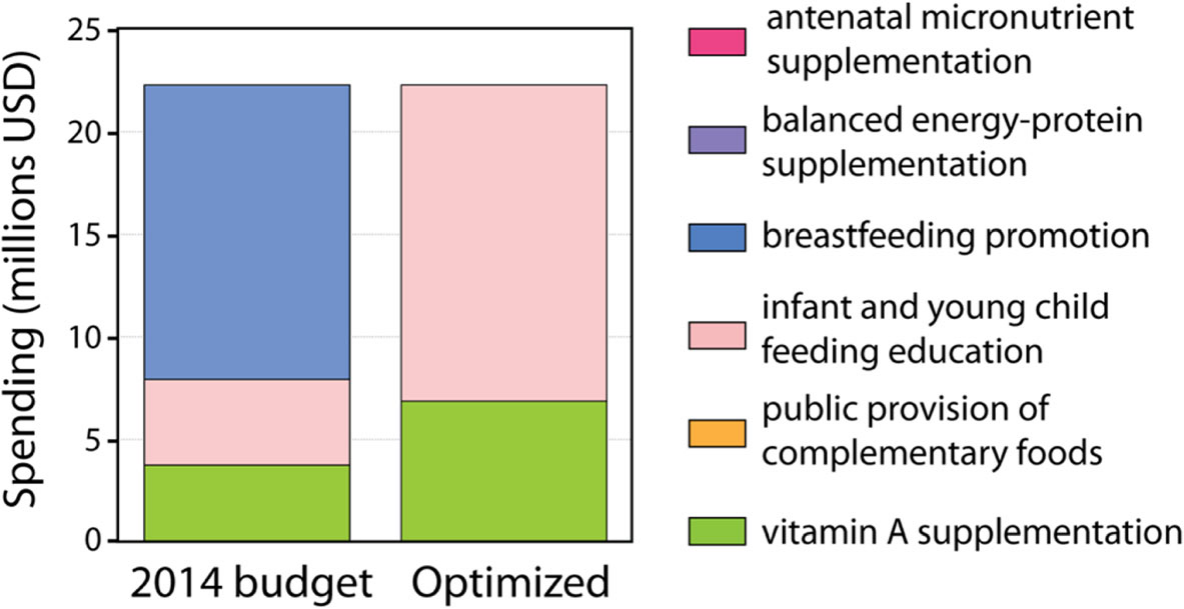
Estimated 2014 allocation and optimal annual allocation across nutrition-specific interventions with budget fixed to 2014 levels. Optimization is with respect to maximizing the number of children not stunted at age 5 years over the 15-year period from 2016 until 2030

Here we present the results of the case study for Bangladesh that was carried out to demonstrate the use of Optima Nutrition. Currently, no published estimates of expenditure on the nutrition-specific interventions in Bangladesh exist. Estimates of nutrition-related expenditure are available in annual reports from the Ministry of Food, the Food Planning and Monitoring Unit (FPMU) [54]. However, those figures do not disaggregate expenditure by specific interventions. Therefore, the current expenditure on the six interventions included in the model was approximated by multiplying the unit cost of each intervention by the current number of beneficiaries covered by that intervention. It needs to be noted that this is a rough estimate. Based on this approach, current annual expenditure on the six interventions included in the analyses was estimated to total ~US$22 million (based on 2014 data). This equates to just $0.75 per person in need. Funding was allocated to breastfeeding promotion (64%), promotion of IYCF for children aged 6–23 months (19%) and vitamin A supplementation (17%) (Fig. 3). Our analyses at the national level suggest that the alive and not stunted objective could be maximized by shifting allocations to spend a majority (69%) on promotion of IYCF for children aged 6–23 months and the remainder (31%) on vitamin A supplementation. With the same level of resources this programmatic shift would be expected to increase the number of children in Bangladesh living without stunting after age 5 years by 1.37 million cumulatively by 2030, an extra ~ 5% compared to continuation of the status quo. Excluding external determinants of reduced stunting, this reallocation of the little available resourcing could reduce national stunting prevalence from 36% to 32% in 2030.

**Fig. 4:**
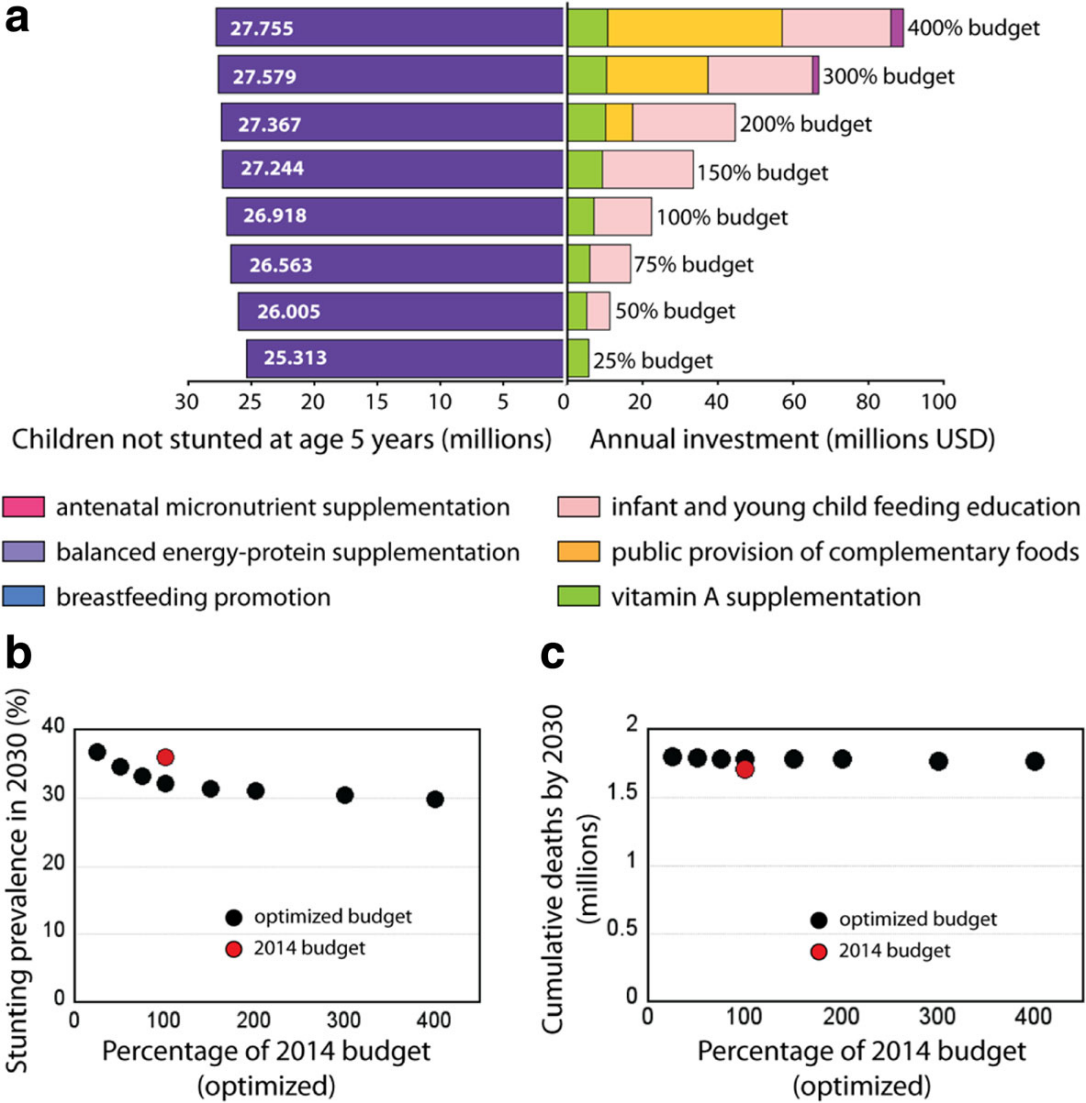
Estimated and optimal annual nutrition-specific spending and corresponding outcomes for a series of budget scenarios. Budget scenarios range from 25% to 400% of the current estimated national nutrition-specific spending in Bangladesh. **a** Interventions included in increasing budgets and their effect on the number of children reaching the age of 5 not stunted by 2030; **b** Overall stunting prevalence (in all age groups) in 2030 under increasing optimized budget; **c** Cumulative number of deaths from 2016 to 2030

The Optima model also allows the user to identify the optimal mix of interventions as the available resource envelope expands. This function of the model is particularly useful because it permits to determine at which point of the envelope expansion new interventions could be added or funded to maximize the program impact on stunting-free survival. For illustration purposes, we calculated the optimal allocation of resources across interventions assuming that the current financing for the six nutritional interventions will expand from the current level to 150%, 200%, 300%, and 400% of the current estimated budget (Fig. 4a). If more resources become available, then IYCF should continue to receive funding until it eventually reaches relatively high coverage and where the marginal cost becomes relatively high (occurring at ~ 150% of the current budget). Once these services are financed, public provision of complementary foods should be the next intervention to be included, followed by antenatal micronutrient supplementation (at around 200% extra total budget). If the total national budget for nutrition were quadrupled and optimally allocated, over 2.2 million more children would reach age 5 years without stunting, and stunting prevalence would drop to below 30% by 2030 (Fig. 4b).

If one aims to optimize resources against different objectives then there will be trade-offs in terms of the outcomes related to the objectives. For example, an objective which minimizes stunting may result in greater numbers of deaths than an objective which is solely focused on minimizing deaths. Our analyses reveal these trade-offs ((Fig. 4c). The effect of increased deaths by maximizing children remaining alive and not stunted is small (77,000) compared to the increased number of averted cases of stunting (1.4 million) if one was to minimize mortality.

If greater budgets were available to provide greater coverage of interventions, then further improvements in the number of children remaining alive and not stunted would be possible. However, the incremental gain per additional dollar spent decreases (Fig. 4a). Sustainable Development Goal 2 calls for a 40% reduction of the number of stunted children by 2025, which if adopted at the country-level for Bangladesh would roughly mean reducing prevalence to below 21.5%. Our analysis suggests that this goal cannot be reached even with a 4-fold increase in spending on these six interventions, even when these resources are spent most cost-effectively. Therefore, to reach this goal at the national level, expansion of other types of interventions such as water, sanitation and hygiene (WASH) [55] and maternal education [56] as well as the crucial underlying economic and development factors would be necessary.

**Fig. 5:**
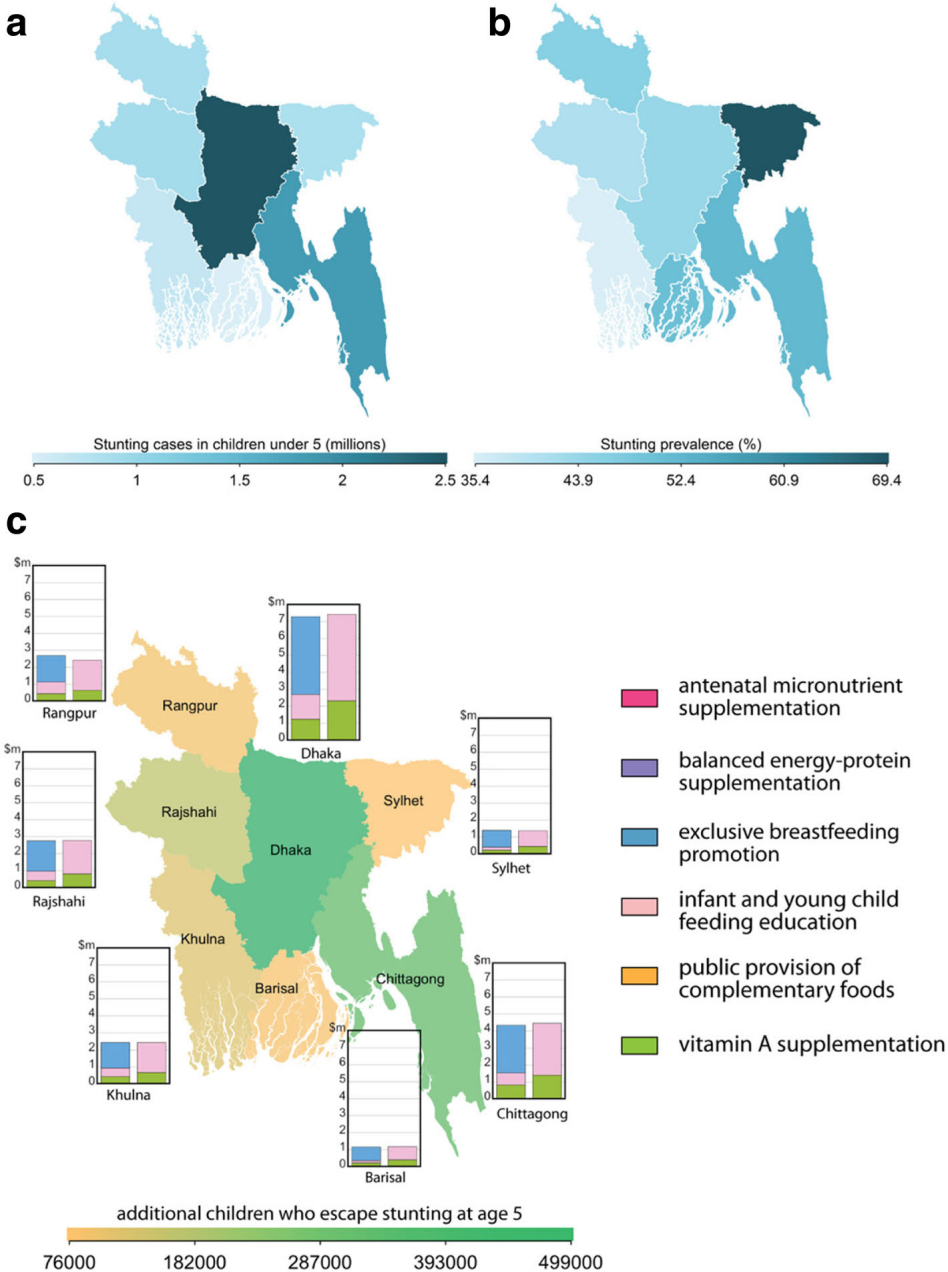
Map of the divisions of Bangladesh, color-coded by **a** total stunting cases in children younger than 5 years; **b** prevalence of stunting among children younger than 5 years; **c** additional children who remain not stunted and alive at age 5 years through an optimal allocation of resources along with the estimated optimal redistribution of resources (2014 spending (left bar) compared to the optimized for the division (right bar)). Image produced by the authors

Optima Nutrition can be used to not only identify the broad program areas for prioritization at a national level given a specific resource envelope, but to also identify priority geographical or administrative regions for targeting. In Bangladesh, Dhaka has the highest number of stunted children (around 2.5 million), whereas the southern regions of Barisal and Khulna have the least (Fig. 5a). However, the highest prevalence of stunting is in Sylhet where around 70% of children in the oldest age group are stunted compared with around 35% in Khulna (Fig. 5b). The current coverage levels of each intervention vary by region (Fig. 5c). Sylhet receives the lowest combined coverage of the interventions and has the highest fraction of the population under poverty line, both of which may account for its higher burden of stunting prevalence. In practice, given the already low financing of nutritional interventions overall we would not recommend reducing funding from any region. However, for illustration purposes we found that if the current available funding in Bangladesh were to be optimally reallocated geographically and by intervention area then it would be possible to have additional impact on the number of children who are living and not stunted by age 5 years. Specifically, compared to the status quo allocation, by 2030 improved allocative efficiency could increase the number of non-stunted children alive at age 5 years by: (i) 1.32 million (5.1%) by broad national program funding allocations; (ii) by 1.36 million (5.3%) if geographical targeting was also included (Fig. 5c). Improved outcomes could be achieved by slightly more prioritization to Dhaka and Chittagong at the expense of Rangpur (Fig. 5c). Similar analyses could be conducted for different levels of resources to guide resource allocations to target the right programs in the right places for greatest impact.

## Discussion

Over the past decade, a convincing investment case for nutrition has been made. The existing literature has explored the cost and impact of expanding high-impact evidence-based interventions [9, 52, 53] and produces estimates of economic impact showing that investing in nutrition in general and in stunting reduction in particular produces economic returns that outweigh the cost of interventions several-fold [57, 58].

While this investment case has been critical in bringing nutrition back to the forefront of the international development agenda, it has been, at the same time, rather general. While it is clear why expanding the coverage of nutrition interventions is needed, little information is available about how such expansion should be carried out in a way that would maximize the impact of every dollar invested.

Decision tools based on mathematical modeling can be useful in informing allocation decisions. Compared to other health areas, such as HIV/AIDS, however, surprisingly few decision-analytic tools for nutrition exist, and none them allows for identifying an optimal allocation of resources across a wide range of nutritional interventions. One group of existing models, including Optifood and the Cost of the Diet (CoD) tool, uses the linear programming approach to develop dietary recommendations under a set of constraints (e.g. affordability, availability of specific foods in a given country context). Optifood has been recently used to develop recommendations for complementary feeding in Indonesia [59], Myanmar [60], and Kenya [61]. Linear programming tools can also be combined with expenditure and consumption data to estimate the proportion of the population that can and cannot afford optimal diets. The CoD has been used in that way to advocate of the expansion of public provision of complementary foods in Indonesia (see [62]). While important, dietary recommendations are not easily translated into specific interventions and the linear programming approach provides no information regarding cost-effectiveness or efficiency of interventions.

The Lives Saved Tool (LiST) has been used in estimating comparative cost-effectiveness of nutrition interventions (e.g. [63, 64]). However, the tool itself is not designed to assess cost-effectiveness directly and the studies mentioned above combined LiST modelling with additional Excel-based models to develop comparative cost effectiveness estimates. The traditional approach to technical assessments of how to achieve the best value for money is to calculate a cost-effectiveness ratio for each intervention in isolation, then rank them in a league table, nominating funding to each intervention in sequence to scale until all resources are depleted. We and others have shown that the traditional league table approach is not the best use of resources, because it does not account for the interactions between interventions and disease burden dynamics due to other implementations [65].

MINIMOD is the only other decision-analytic model that examines allocative efficiency of nutrition investments and identifies a mix of interventions that maximizes specific nutrition or health outcomes. One particular strength of the model is that is allows for comparison of different delivery platforms for a single intervention (e.g. vitamin a supplementation through campaigns, facility-based supplementation, fortification of different food vehicles) [66]. However, currently, the model is limited to micro-nutrient interventions and requires very specific data on consumption, diet composition, and micro-nutrient deficiency levels which need to be collected through specialized surveys (see [67]). Furthermore, MINMOD does not have a user interface which limits its utility for program managers and decision makers. In contrast, Optima nutrition considers a much broader range of interventions, including micronutrient supplementation, behavioural interventions, and provision of complementary foods. Most of the data the model requires is easily available through surveys such as DHS or MICS, of which several waves exist for most developing countries. Finally, a graphic user interface is under development. The final version of the tool will be cloud-based and available online to researchers, program managers and policy makers.

As with any model being applied to a complex real-world problem, applications of the Optima Nutrition tool will have limitations. Mathematical models are simplifications of the processes that they represent. The current application had limitations in model structure. This includes considering the populations in Bangladesh to be uniformly mixed within each age band. Interventions were also considered without flow-on effects; for example, breastfeeding promotion targeted to mothers of children aged < 6 months may have ongoing benefits for age-appropriate breastfeeding up to 23 months, but these effects are likely to be setting- and program-specific and are largely unknown. In particular, details of the content of the programs (e.g. number of visits and help provided during each visit for IYCF) are rarely studied and therefore difficult to quantify. Another type of limitation is around model parameterization and data. Where studies were available which evaluated the impact of nutrition interventions from the literature, they were used in this study, assuming the effectiveness levels were consistent universally, when in reality they are more likely to be country-dependent. This leaves inherent uncertainty in the optimization. In addition, uncertainty in the demographics (e.g. age structure of under-five population), nutritional (e.g. stunting prevalence, feeding practices) and costing (e.g. unit costs of interventions, structure of cost functions) data collected in country surveys contribute to overall uncertainty in the results and conclusions of the study. The results are implications of the data used. We used the best data available, assuming that population surveys (such as DHS) are reasonably precise and representative of the target populations. We used a complementary nutritional interventions costing study in Bangladesh to parameterize the unit costs used in this model. That costing study was performed with an intention to be used in this analytical study. Despite the limitations in the structure and parameterization of the tool, we believe that the approach taken in this study appropriately sheds new insight into prioritization of nutrition interventions in Bangladesh. Modelled outcomes should only serve as guidance in the decision-making process, representing the best available evidence at the time. Other factors, including various social, political and implementation demands and constraints need to be considered.

Currently, Optima nutrition is limited to a set of six nutrition- specific interventions affecting stunting. An extension of the model to other aspects of malnutrition (wasting, anaemia), nutrition behaviours (prevalence of breastfeeding) and nutrition-specific interventions targeting those outcomes is ongoing. The expanded version of Optima Nutrition will also include some nutrition-sensitive interventions such as water and sanitation (WASH) and family planning.

## Conclusions

Reaching the SDG nutrition and food security target will require a systematic effort to maximize the efficiency and impact of nutrition budgets. We hope that Optima Nutrition can be a useful in informing priority setting and allocation of resources across nutritional interventions.

## Abbreviations

AMS: Antenatal micronutrient supplementation
ASD: Adaptive Stochastic Descent
CMAM: Community management of acute malnutrition
CoD: Cost of the Diet
FANTA: Food and Nutrition Technical Assistance
FPMU: Food Planning and Monitoring Unit
IYCF: Improved infant and young child feeding
LiST: Lives Saved Tool
SDGs: Sustainable development goals
WASH: Water, sanitation and hygiene
WBCi: World Breastfeeding Costing Initiative

## References

[1] De Onis M, Blössner M, Borghi E (2010). Global prevalence and trends of overweight and obesity among preschool children. Am J Clin Nutr.

[2] Black RE, Victora CG, Walker SP, Bhutta ZA, Christian P, De Onis M, Ezzati M, Grantham-McGregor S, Katz J, Martorell R (2013). Maternal and child undernutrition and overweight in low-income and middle-income countries. Lancet.

[3] Grantham-McGregor SM, Powell CA, Walker SP, Himes JH (1991). Nutritional supplementation, psychosocial stimulation, and mental development of stunted children: the Jamaican study. Lancet.

[4] Alderman H, Hoddinott J, Kinsey B (2006). Long term consequences of early childhood malnutrition. Oxf Econ Pap.

[5] Walker SP, Chang SM, Powell CA, Simonoff E, Grantham-McGregor SM (2007). Early childhood stunting is associated with poor psychological functioning in late adolescence and effects are reduced by psychosocial stimulation. J Nutr.

[6] Kar BR, Rao SL, Chandramouli B (2008). Cognitive development in children with chronic protein energy malnutrition. Behav Brain Funct.

[7] Victora CG, Adair L, Fall C, Hallal PC, Martorell R, Richter L, Sachdev HS, for the maternal and child undernutrition study group (2008). Maternal and child undernutrition: consequences for adult health and human capital. Lancet.

[8] The consequences of early childhood growth failure over the life course. http://ebrary.ifpri.org/utils/getfile/collection/p15738coll2/id/124899/filename/124900.pdf.

[9] Bhutta ZA, Das JK, Rizvi A, Gaffey MF, Walker N, Horton S, Webb P, Lartey A, Black RE, The Lancet Nutrition Interventions Review Group (2013). Evidence-based interventions for improvement of maternal and child nutrition: what can be done and at what cost?. Lancet.

[10] Victora CG, de Onis M, Hallal PC, Blössner M, Shrimpton R (2010). Worldwide timing of growth faltering: revisiting implications for interventions. Pediatrics.

[11] Kramer MS, Kakuma R (2012). Optimal duration of exclusive breastfeeding. Cochrane Database Syst Rev.

[12] Espo M, Kulmala T, Maleta K, Cullinan T, Salin ML, Ashorn P (2002). Determinants of linear growth and predictors of severe stunting during infancy in rural Malawi. Acta Paediatr.

[13] Saha KK, Frongillo EA, Alam DS, Arifeen SE, Persson LA, Rasmussen KM (2008). Appropriate infant feeding practices result in better growth of infants and young children in rural Bangladesh. Am J Clin Nutr.

[14] Arimond M, Ruel MT (2004). Dietary diversity is associated with child nutritional status: evidence from 11 demographic and health surveys. J Nutr.

[15] Steyn NP, Nel JH, Nantel G, Kennedy G, Labadarios D (2006). Food variety and dietary diversity scores in children: are they good indicators of dietary adequacy?. Public Health Nutr.

[16] Onyango AW, Borghi E, de Onis M, Casanovas Mdel C, Garza C (2014). Complementary feeding and attained linear growth among 6-23-month-old children. Public Health Nutr.

[17] Dewey KG, Adu-Afarwuah S (2008). Systematic review of the efficacy and effectiveness of complementary feeding interventions in developing countries. Matern Child Nutr.

[18] Fink G, Gunther I, Hill K (2011). The effect of water and sanitation on child health: evidence from the demographic and health surveys 1986-2007. Int J Epidemiol.

[19] D'Alimonte MR, Rogers H, de Ferranti D, Shekar M, Kakietek J, Dayton Eberwein J, Walters D (2017). Financing the global nutrition targets. An investment framework for nutrition.

[20] de Savigny D, Casale H, Mbuya C, Reid G (2008). Fixing health systems.

[21] Knaul FM, González-Pier E, Gómez-Dantés O, García-Junco D, Arreola-Ornelas H, Barraza-Lloréns M, Sandoval R, Caballero F, Hernández-Avila M, Juan M (2012). The quest for universal health coverage: achieving social protection for all in Mexico. Lancet.

[22] Bitran R (2013). Explicit health guarantees for Chileans: the AUGE benefits package. UNICO Studies Series 21.

[23] Winfrey W, McKinnon R, Stover J (2011). Methods used in the lives saved tool (LiST). BMC Public Health.

[24] Walker N, Tam Y, Friberg IK (2013). Overview of the lives saved tool (LiST). BMC Public Health.

[25] Heymann H (2012). PROFILES: an evidence-based tool for nutrition education and promotion. J Nutr Educ Behav.

[26] World Breastfeeding Costing Initiative (WBCi). [http://ibfan.org/world-breastfeeding-costing-initiative]

[27] CMAM Costing Tool. [http://www.fantaproject.org/tools/cmam-costing-tool]

[28] Brown KH, Engle-Stone R, Kagin J, Rettig E, Vosti SA (2015). Use of optimization modeling for selecting National Micronutrient Intervention Strategies: an example based on potential programs for control of vitamin a deficiency in Cameroon. Food Nutr Bull.

[29] FANTA III. Food and Nutrition Technical Assistance. [http://www.fantaproject.org/]

[30] Kerr CC, Stuart RM, Gray RT, Shattock AJ, Fraser-Hurt N, Benedikt C, Haacker M, Berdnikov M, Mahmood AM, Jaber SA (2015). Optima: a model for HIV epidemic analysis, program prioritisation, and resource optimisation. JAIDS.

[31] The World Bank (2015). Optimizing HIV Investments in Armenia.

[32] Grantham K, Reagan D, Law M, Wilson DP (2016). Optimizing Investments in the National HIV responses of Indonesia and Thailand: a report for World Health Organization South-East Asia regional office.

[33] Masaki E, Fraser N, Haacker M, Obst M, Wootton R, Sunkutu R, Gorgens M, Gray RT, Shattock A, Kerr CC (2015). Zambia's HIV response: prioritised and strategic allocation of HIV resources for impact and sustainability.

[34] Kelly S, Shattock A, Kerr CC, Gama T, Nhlabatsi N, Zagatti G, Harimurti P, Wilson DP, Gorgens M (2014). HIV mathematcal modelling to support Swaziland’s development of its HIV investment case.

[35] Scott N, Hussain SA, Martin-Hughes R, Fowkes FJ, Kerr CC, Pearson R, Kedziora DJ, Killedar M, Stuart RM, Wilson DP (2017). Maximizing the impact of malaria funding through allocative efficiency: using the right interventions in the right locations. Malar J.

[36] Black RE, Allen LH, Bhutta ZA, Caulfield LE, de Onis M, Ezzati M, Mathers C, Rivera J, for the maternal and child undernutrition study group (2008). Maternal and child undernutrition: global and regional exposures and health consequences. Lancet.

[37] Lamberti LM, Walker CLF, Noiman A, Victora C, Black RE (2011). Breastfeeding and the risk for diarrhea morbidity and mortality. BMC Public Health.

[38] Wang H, Liddell CA, Coates MM, Mooney MD, Levitz CE, Schumacher AE, Apfel H, Iannarone M, Phillips B, Lofgren KT (2014). Global, regional, and national levels of neonatal, infant, and under-5 mortality during 1990–2013: a systematic analysis for the Global Burden of Disease Study 2013. Lancet.

[39] Haider BA, Bhutta ZA (2012). Multiple-micronutrient supplementation for women during pregnancy. Cochrane Database Syst Rev.

[40] Imdad A, Bhutta ZA (2011). Effect of balanced protein energy supplementation during pregnancy on birth outcomes. BMC Public Health.

[41] Imdad A, Yakoob MY, Bhutta ZA (2011). Effect of breastfeeding promotion interventions on breastfeeding rates, with special focus on developing countries. BMC Public Health.

[42] Imdad A, Yakoob MY, Bhutta ZA (2011). Impact of maternal education about complementary feeding and provision of complementary foods on child growth in developing countries. BMC Public Health.

[43] Lassi ZS, Das JK, Zahid G, Imdad A, Bhutta ZA (2013). Impact of education and provision of complementary feeding on growth and morbidity in children less than 2 years of age in developing countries: a systematic review. BMC Public Health.

[44] Imdad A, Herzer K, Mayo-Wilson E, Yakoob MY, Bhutta ZA (2010). Vitamin a supplementation for preventing morbidity and mortality in children from 6 months to 5 years of age. Cochrane Database Syst Rev.

[45] Haider BA, Bhutta ZA. Neonatal vitamin a supplementation for the prevention of mortality and morbidity in term neonates in developing countries. Cochrane Database Syst Rev. 2011;(10):CD006980. 10.1002/14651858.CD006980.pub2. 10.1002/14651858.CD006980.pub2 21975758

[46] Kerr CC, Smolinski T, Dura-Bernal S, Wilson D (2017). Optimization by Bayesian adaptive locally linear stochastic descent.

[47] International Food Policy Research Institute: Global nutrition report 2016: from promise to impact: ending malnutrition by 2030. vol. 2016. Washington DC: International Food Policy Research Institute; 2016.

[48] Olofin I, McDonald CM, Ezzati M, Flaxman S, Black RE, Fawzi WW, Caulfield LE, Danaei G, for the Nutrition Impact Model Study (anthropometry cohort pooling) (2013). Associations of suboptimal growth with all-cause and cause-specific mortality in children under five years: a pooled analysis of ten prospective studies. PLoS One.

[49] Institute of Statistical Research and Training, Bangladesh Bureau of sStatistics (2015). Population projection of Bangladesh: dynamics and trends 2011–2061.

[50] National Institute of Population Research and Training (NIPORT), Mitra and Associates, ICF International (2016). Bangladesh demographic and health survey 2014.

[51] National Institute of Population Research and Training (NIPORT), Mitra and Associates, ICF International (2013). Bangladesh demographic and health survey 2011.

[52] Shekar M, Kakietek J, Dayton Eberwein J, Walters D (2017). An investment framework for nutrition: reaching the global targets for stunting, anemia, breastfeeding, and wasting.

[53] Horton S, Shekar M, McDonald C, Mahal A, Brooks JK (2010). Scaling up nutrition: What will it cost?.

[54] Food Planning and Monitoring Unit (2015). National Food Policy plan of action and country investment plan monitoring report 2015.

[55] Cumming O, Cairncross S (2016). Can water, sanitation and hygiene help eliminate stunting? Current evidence and policy implications. Matern Child Nutr.

[56] Headey D, Hoddinott J, Ali D, Tesfaye R, Dereje M (2015). The other Asian enigma: explaining the rapid reduction of undernutrition in Bangladesh. World Dev.

[57] Hoddinott J, Alderman H, Behrman JR, Haddad L, Horton S (2013). The economic rationale for investing in stunting reduction. Matern Child Nutr.

[58] Alderman H, Haddad L, Headey DD, Smith L (2014). Association between economic growth and early childhood nutrition. Lancet Glob Health.

[59] Fahmida U, Santika O, Kolopaking R, Ferguson E (2014). Complementary feeding recommendations based on locally available foods in Indonesia. Food Nutr Bull.

[60] Hlaing LM, Fahmida U, Htet MK, Utomo B, Firmansyah A, Ferguson EL (2016). Local food-based complementary feeding recommendations developed by the linear programming approach to improve the intake of problem nutrients among 12-23-month-old Myanmar children. Br J Nutr.

[61] Vossenaar M, Knight FA, Tumilowicz A, Hotz C, Chege P, Ferguson EL (2017). Context-specific complementary feeding recommendations developed using Optifood could improve the diets of breast-fed infants and young children from diverse livelihood groups in northern Kenya. Public Health Nutr.

[62] Baldi G, Martini E, Catharina M, Muslimatun S, Fahmida U, Basuni Jahari A, Hardinsyah, Frega R, Geniez P, Grede N (2013). Cost of the diet (CoD) tool: first results from Indonesia and applications for policy discussion on food and nutrition security. Food Nutr Bull.

[63] Shekar M, Dayton-Eberwein J, Kakietek J (2016). The cost of stunting in South Asia and the benefits of public Investments in Nutrition.

[64] Kakietek J, Shekar M, Dayton Eberwein J, Walters D, Shekar M, Kakietek J, Dayton Eberwein J, Walters D (2017). Financing needs to reach the four global nutrition targets: stunting, anemia, breastfeeding, and wasting. An investment framework for nutrition: reaching the global targets for stunting, anemia, breastfeeding, and wasting.

[65] Chiu C, Johnson LF, Jamieson L, Larson BA, Meyer-Rath G (2017). Designing an optimal HIV programme for South Africa: Does the optimal package change when diminishing returns are considered?. BMC Public Health.

[66] Vosti SA, Kagin J, Engle-Stone R, Brown KH (2015). An economic optimization model for improving the efficiency of vitamin a interventions: an application to young children in Cameroon. Food Nutr Bull.

[67] Engle-Stone R, Nankap M, Ndjebayi AO, Vosti SA, Brown KH (2015). Estimating the effective coverage of programs to control vitamin a deficiency and its consequences among women and young children in Cameroon. Food Nutr Bull.

