# Correction to**:** Optima nutrition: an allocative efficiency tool to reduce childhood stunting by better targeting of nutrition-related interventions

**DOI:** 10.1186/s12889-018-5409-6

**Published:** 2018-04-26

**Authors:** Ruth Pearson, Madhura Killedar, Janka Petravic, Jakub J. Kakietek, Nick Scott, Kelsey L. Grantham, Robyn M. Stuart, David J. Kedziora, Cliff C. Kerr, Jolene Skordis-Worrall, Meera Shekar, David P. Wilson

**Affiliations:** 1 Burnet Institute, Melbourne, Australia; 2 Department of Epidemiology and Preventive Medicine, Monash University, Melbourne, Australia; 3 Optima Consortium for Decision Science, Melbourne, Australia; 4 The World Bank, ICF International, Washington D.C., USA; 5 Department of Mathematical Sciences, University of Copenhagen, Copenhagen, Denmark; 6 Complex Systems Group, School of Physics, University of Sydney, Sydney, Australia; 7 Institute for Global Health, University College London, London, UK

## Correction to: BMC Public Health (2018) 18:384 DOI: 10.1186/s12889-018-5294-z

It has been highlighted that the original manuscript [1] contains a typesetting error in the name of Meera Shekar. This had been incorrectly captured as Meera Shekhar in the original article which has since been updated.
